# Vereinbarkeit von Schwangerschaft, Elternschaft, Ärztlicher Weiterbildung und Karriere in der Radiologie

**DOI:** 10.1007/s00117-024-01379-w

**Published:** 2024-10-29

**Authors:** Eva See, Christian Herold, Fabian Bamberg, Alexandra Ljimani

**Affiliations:** 1 Abteilung Radiologie, Hochwaldkrankenhaus Bad Nauheim, Gesundheitszentrum Wetterau gGmbH, Chaumontplatz 1, 61231 Bad Nauheim, Deutschland; 2 Universitätsklinik für Radiologie und Nuklearmedizin, Universitätsklinikum AKH Wien, Wien, Österreich; 3 Klinik für Diagnostische und Interventionelle Radiologie, Universitätsklinikum Freiburg, Freiburg, Deutschland; 4 Institut für Diagnostische und Interventionelle Radiologie, Heinrich-Heine-Universität, Medizinische Fakultät, Moorenstr. 5, 40225 Düsseldorf, Deutschland

In Zeiten von demografischem Wandel und Fachkräftemangel steigt der Bedarf an qualifizierten Ärztinnen und Ärzten. Um diesen Anforderungen gerecht zu werden, ist es notwendig, dass auch Mütter und Väter den fachärztlichen Status erreichen und ihre Karriere in der klinischen Versorgung fortsetzen. Hier gilt es, Strukturen an die Bedürfnisse eines modernen Familienlebens mit geteilter Sorgearbeit und Erwerbstätigkeit anzupassen.

## Rolle der Radiologie

Die Radiologie, als wichtiges Schnittstellenfach, ist geprägt durch eine spannende klinische sehr interdisziplinäre Tätigkeit über das gesamte Spektrum der Medizin hinweg. Die bildgestützte Diagnostik und Therapie und hoher technischer Fortschritt resultieren in einer anspruchsvollen ärztlichen Weiterbildung des Faches Radiologie. Dies führt zu hoher Arbeitslast der regulären Arbeitszeiten, oft gepaart mit der Notwendigkeit des kontinuierlichen lebenslangen Lernens, was die Möglichkeit, Beruf und Familie in Einklang zu bringen, deutlich erschwert. Besonders Frauen sind hier zusätzlich zu den biologischen Voraussetzungen durch traditionelle Rollenzuweisungen oft mehrfach beansprucht. Allerdings bietet das Fach Radiologie exzellente strukturelle Voraussetzungen für einen Wandel hin zu einer verbesserten Vereinbarkeit von Familie und Beruf und sollte daher als Vorbild für andere Fachdisziplinen agieren.

## Stellenwert eines unterstützenden Umfeldes

Neben einer möglichen Unterstützung im privaten Umfeld und unter Voraussetzung einer Betreuungsmöglichkeit ist die Unterstützung durch Kolleginnen und Kollegen, Vorgesetzte und Arbeitgebende entscheidend für eine erfolgreiche Vereinbarkeit von Familie und Beruf. Kollegiale Rücksichtnahme und eine offene Kommunikation über die Bedürfnisse sollten in jedem radiologischen Team das oberste Gebot sein und ist eine signifikante Komponente für ein positives Arbeitsklima. Insbesondere im Hinblick auf Schwangere sowie Kolleginnen und Kollegen in und nach der Elternzeit ist diese kollegiale Rücksichtnahme besonders wichtig. Vorgesetzte und Arbeitgebende sollten sich zudem ihrer Vorbildfunktion bewusst sein und aktiv für familienfreundliche Strukturen eintreten. Beispielsweise sollte in einem wertschätzenden Miteinander der Fokus daraufgelegt werden, die Ressourcen und Expertise, die Schwangere und Eltern erbringen, zu betonen und anzuerkennen.

## Netzwerke und Mentoring

**Abb. 1:**
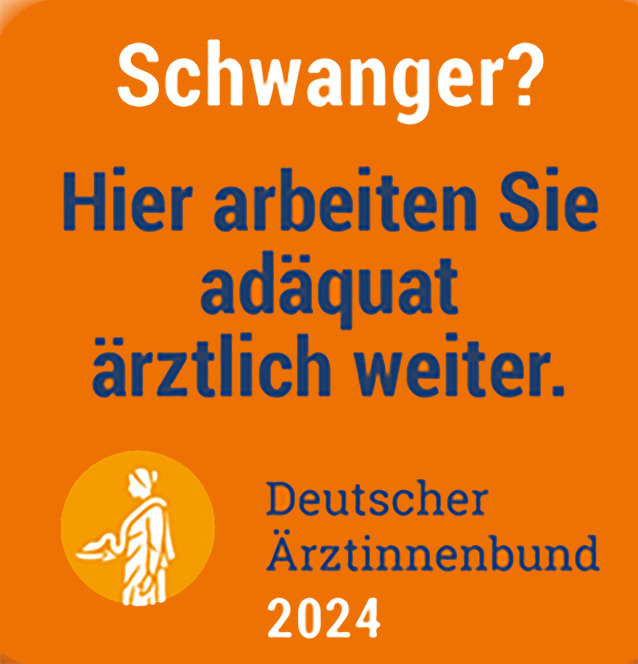
Deutscher Ärztinnenbund-Siegel Mutterschutz. (© DÄB/Anne-Claire Martin. Alle Rechte vorbehalten)

Austausch mit anderen Kolleginnen und Kollegen in ähnlichen Lebenssituationen kann wertvolle Unterstützung bieten. Netzwerke und Mentoring-Programme, wie das der *Radiologinnen@DRG* und *Eltern@DRG* fördern den Erfahrungsaustausch, Wissenstransfer und emotionale Unterstützung. Weitere wichtige Beratungsstellen bietet der Marburger Bund, der − neben einer rechtlichen Beratung für Mitglieder und einer Online-Positivliste von Arbeitgebenden − im Rahmen der Tarifverhandlungen für den TV‑Ä der Unikliniken Hessen 2023 eine Clearingstelle zum Arbeiten in der Schwangerschaft eingerichtet hat. Eine weitere wichtige Initiative, um die Bedeutung dieser Thematik hervorzuheben, ist das durch den Deutschen Ärztinnenbund (DÄB) verliehene *Siegel Mutterschutz,* das Arbeitgebende auszeichnet (Abb. 1), welche schwangeren Ärztinnen gute Möglichkeiten bieten, sicher weiterzuarbeiten und ihren Karriereweg trotz Familienplanung nicht unterbrechen zu müssen. Der DÄB begründete zudem den fächerübergreifenden Workshop Mutterschutz der Ärztekammer Nordrhein, welcher ein bislang unveröffentlichtes Positionspapier zum Arbeiten in Schwangerschaft und Stillzeit erarbeitet hat, das aktuell den Prozess für eine AWMF-Leitlinie durchläuft. Auch das Bündnis Junge Ärztinnen und Ärzte setzt sich im Positionspapier zur ärztlichen Weiterbildung anlässlich der Krankenhausreform für eine Förderung von Schwangeren und Eltern ein (BJÄ PP). Als Ergebnis forderte der 128. Deutsche Ärztetag 2024 (DÄT2024) in zwei Beschlussanträgen, Rahmenbedingungen zur Verbesserung der Vereinbarkeit von ärztlicher Erwerbstätigkeit und Weiterbildung mit unbezahlter Sorgearbeit in der Familie gesetzlich zu verankern [Ic – 90, IIId-04]. Dies ist ein wichtiges Signal zur tatsächlichen Umsetzung wichtiger struktureller Veränderungen an die jüngeren und nachfolgenden Generationen. Die wichtige Verbindung zwischen diesen theoretischen Forderungen und der praktischen Umsetzung im Arbeitsumfeld hin zu einer gelungenen Kombination von Familie und Beruf liefern schließlich Vorbilder beider Geschlechter in den einzelnen Teams. Sie sind am Ende der entscheidende Faktor für eine individuell erfolgreiche Vereinbarkeit für die einzelnen Betroffenen vor Ort.

## Schwangerschaft, Elternzeit und Stillzeit

Der DÄT2024 hat sich für die Fortführung ärztlicher Tätigkeit (werdender) Mütter und Väter, insbesondere während der Zeit ihrer Weiterbildung, ausgesprochen. Primäres Ziel ist der Erhalt der ärztlichen Arbeitskraft sowie das schnellstmögliche Erreichen der Fachärztinnen- bzw. Facharztreife. Hierfür ist eine positive Auslegung des Mutterschutzgesetzes (MuSchG) für schwangere und stillende Ärztinnen essenziell. Dies beinhaltet eine flächendeckende Erstellung von Gefährdungsbeurteilungen und Ermöglichungsbeschreibungen an jedem Arbeitsplatz, im Idealfall als Standard Operating Procedure (SOP) gemäß dem Workshop Mutterschutz der Ärztekammer Nordrhein. Für die Radiologie kann hierfür eine Positivliste bei der Deutschen Röntgengesellschaft e. V. angefragt werden. Sofern sich die schwangere Ärztin in Weiterbildung befindet, sollte gemäß Empfehlung des Workshop Mutterschutz zudem die Erstellung eines aktualisierten Rotationsplans für die Zeit während und nach der Schwangerschaft bzw. nach Mutterschutz/Elternzeit mit dem Ziel eines möglichst raschen Vorankommens in der ärztlichen Weiterbildung enthalten sein (Positionspapier WM).

Die Umgestaltung von Arbeitsplätzen und Tätigkeitsfeldern soll der schwangeren Mitarbeiterin die Fortführung ihrer Weiterbildung und/oder Fortbildungen sowie die Ausübung ihres Berufes ermöglichen.

Eine kontinuierliche Beschäftigung auch in veränderten Lebenssituationen wie Schwangerschaft, Elternschaft, aber auch Krankheit dient hierbei sowohl der Kompetenzsteigerung als auch dem Kompetenzerhalt beider Geschlechter. In der Novellierung des TV‑Ä (§ 3a Absatz 6) wird zudem Eltern fortan Unterstützung bei abteilungsinternen Fort- und Weiterbildungsmaßnahmen sowie externen Fortbildungs- und Qualifizierungsmaßnahmen analog den für Ärztinnen und Ärzten, die sich nicht in Elternzeit befinden eingeräumt (Quelle: Tarifvertrag).

## Beruflicher Wiedereinstieg nach der Geburt oder Elternzeit

Die aus den Tarifverhandlungen des TV‑Ä der Unikliniken Hessen 2023 hervorgegangene Clearingstelle zum Arbeiten in der Schwangerschaft hat weitere Eckpunkte für den Wiedereinstieg nach Elternzeit erarbeitet, welche in der aktuellen Tarifrunde 2024 in die Novellierung des TV‑Ä (§ 3a) integriert wurden (Quelle: Tarifvertrag). Hierzu gehörten z. B. mindestens ein protokolliertes Wiedereinstiegsgespräch, bei welchem individuell notwendige Rahmenbedingungen der Tätigkeit wie Teilzeit etc. festgelegt werden und bei welchem auch eine weitere Person der Wahl anwesend sein kann, oder die Möglichkeit zur Mitarbeit vor dem Wiedereinstieg, um die berufliche Qualifikation aufrechtzuerhalten.

## Weitere Schlüsselfaktoren eines notwendigen strukturellen Wandels [Ic – 90]

1. *Flächendeckender Ausbau der Kinderbetreuung:* Kliniken und Radiologie-Praxen sollten in die Lage versetzt werden, betriebliche Kindergärten/abteilungseigene Kinderbetreuung zu organisieren und/oder Kooperationen mit nahegelegenen Betreuungseinrichtungen einzugehen. Dies würde Eltern ermöglichen, ihre Kinder in der Nähe ihres Arbeitsortes betreuen zu lassen, was in bestimmten Konstellationen Zeit und Zeitdruck reduziert. Nicht nur im Fall einer größeren Distanz zwischen Wohn- und Arbeitsort kann ein wohnortnaher Betreuungsplatz jedoch individuell sinnvoller sein, um die Arbeitszeiten eines bestimmten Elternteils von den Betreuungszeiten zu entkoppeln und allen Bezugspersonen Sorgearbeit zu ermöglichen. Dies verdeutlicht, dass ein umfassender Ausbau der Kinderbetreuung unerlässlich ist.
  Ein gesetzlicher Anspruch auf Notfallbetreuung in Klinik- oder Betriebskindergärten ist zudem dringend notwendig. Dies sollte auch für Kinder gelten, die bereits in öffentlichen oder privaten Kindergärten angemeldet sind. Solche Notfalllösungen helfen, kurzfristige private Betreuungslücken in der regulär geplanten Arbeitszeit zu schließen und/oder die Eltern dazu zu befähigen, im Notfall spontan Dienste übernehmen zu können.
2. *Flexible Arbeitszeiten:* Kliniken und Praxen sollten verschiedene Voll- und Teilzeitmodelle anbieten, die den individuellen Bedürfnissen der Mitarbeiterinnen und Mitarbeiter gerecht werden. Flexibilität bei Dienstplänen und die Möglichkeit, Arbeitszeiten an private Rahmenbedingungen anzupassen, sind entscheidend.
3. *Heimarbeitsplätze: *Entgegen vielen anderen Disziplinen in der Medizin, besteht in der Radiologie die Möglichkeit, von zu Hause aus zu arbeiten, welches als Herausstellungsmerkmal entsprechend gefördert werden sollte. Heimarbeitsplätze ermöglichen es Eltern, ihre Arbeitszeit um die Zeit für potenzielle Arbeitswege auszuweiten und gleichzeitig ihre Arbeit zeitlich flexibel zu gestalten, um diese besser auf den Tagesablauf als Familie abzustimmen. Neben der diagnostischen Befundung oder der Lehre und Weiterbildung besteht auch nach arbeitsreichen Diensten oder im Krankheitsfall von Angehörigen durch Heimarbeitsplätze die Möglichkeit, Arbeitskraft sinnvoll zu erhalten.
4. *Geteilte Führungspositionen:* Das Angebot von geteilten Führungspositionen auf oberärztlicher und chefärztlicher Ebene sollte als moderne Führungsmethode anerkannt und gefördert werden. Diese Modelle ermöglichen es, Führungsverantwortung zu übernehmen, ohne die volle zeitliche Belastung alleine tragen zu müssen. Dies kann nicht nur die Karrierechancen für Mütter und Väter erheblich verbessern, sondern auch in vielen weiteren Arbeits- und Lebenssituationen von Vorteil sein (z. B. längerer Krankheit, Behinderung, Weiterbildung, Lehrtätigkeit, Ehrenamt). Kliniken und Praxen würden dadurch das verfügbare Spektrum an Fähigkeiten und Erfahrungen deutlich erweitern.

Deutlich ist, dass radiologische Kliniken und Praxen die genannten Möglichkeiten hin zu einer verbesserten Vereinbarkeit von Schwangerschaft, Elternschaft, ärztlicher Weiterbildung und Karriere in der Radiologie implementieren und fördern sollten. Ergänzend zur spannenden klinischen Tätigkeit in einem hochinnovativen Umfeld in der Radiologie, erhöht sich dadurch die Attraktivität unseres Fachgebiets signifikant. Dies sind Schlüsselkomponenten, um Fachkräfte zu halten und nachfolgende Generationen für die Radiologie zu begeistern. Beides ist notwendig, um den stetig wachsenden Herausforderungen heute und in Zukunft zu begegnen und somit weiterhin einen herausragenden positiven Beitrag zur medizinischen Versorgung leisten zu können.

## Fazit für die Praxis

- *Vorteil des Fachgebiets Radiologie:* Gute strukturelle Voraussetzungen für einen Wandel hin zu einer verbesserten Vereinbarkeit von Familie und Beruf.
- *Unterstützendes Arbeitsumfeld:* Förderung von kollegialer Rücksichtnahme, Wertschätzung und offener Kommunikation.
- *Netzwerke und Mentoring:* Unterstützung und Ausbau von Netzwerken sowie Mentoring-Programmen für Eltern (z. B. *Radiologinnen@DRG* und *Eltern@DRG*). Vorbilder für eine gelungene Kombination von Beruf und Familie in den einzelnen Teams.
- *Ärztliche Weiterbildung:* Primäres Ziel ist der Erhalt der ärztlichen Arbeitskraft sowie das schnellstmögliche Erreichen der Fachärztinnen- bzw. Facharztreife. Hierfür müssen ggf. individualisierte Rotationspläne für die Zeit während der Schwangerschaft sowie Mutterschutz bzw. Elternzeit erstellt werden.
- *Erweiterung der Kinderbetreuung:* Kliniken und Praxen sollten betriebliche Kindergärten einrichten oder Kooperationen mit bestehenden Einrichtungen eingehen. „Abteilungseigene“ Kinderbetreuung ist hier ein wichtiger Stickpunkt. Zudem sollte die flächendeckende Notfallbetreuung für Kinder von medizinischem Personal eingeführt werden.
- *Flexible Arbeitszeitmodelle:* Entwicklung und Implementierung von flexiblen Arbeitszeitmodellen, einschließlich Teilzeit- und Schichtmodellen. Gleitzeiten morgens und abends sind insbesondere in der Radiologie denkbare Ansätze in dieser Hinsicht.
- *Heimarbeitsplätze:* Förderung der Heimarbeit, wo immer möglich (z. B. diagnostische Befundung, administrative Aufgaben, Lehrtätigkeiten, Fortbildungen).
- *Geteilte Führungspositionen:* Etablierung von Modellen für geteilte Führungspositionen, um u. a. die Karrierechancen von Eltern zu verbessern.
